# Spiking Autoencoders With Temporal Coding

**DOI:** 10.3389/fnins.2021.712667

**Published:** 2021-08-13

**Authors:** Iulia-Maria Comşa, Luca Versari, Thomas Fischbacher, Jyrki Alakuijala

**Affiliations:** Google Research, Zürich, Switzerland

**Keywords:** spiking networks, temporal coding, latency coding, backpropagation, autoencoders, inhibition, biologically-inspired artificial intelligence

## Abstract

Spiking neural networks with temporal coding schemes process information based on the relative timing of neuronal spikes. In supervised learning tasks, temporal coding allows learning through backpropagation with exact derivatives, and achieves accuracies on par with conventional artificial neural networks. Here we introduce spiking autoencoders with temporal coding and pulses, trained using backpropagation to store and reconstruct images with high fidelity from compact representations. We show that spiking autoencoders with a single layer are able to effectively represent and reconstruct images from the neuromorphically-encoded MNIST and FMNIST datasets. We explore the effect of different spike time target latencies, data noise levels and embedding sizes, as well as the classification performance from the embeddings. The spiking autoencoders achieve results similar to or better than conventional non-spiking autoencoders. We find that inhibition is essential in the functioning of the spiking autoencoders, particularly when the input needs to be memorised for a longer time before the expected output spike times. To reconstruct images with a high target latency, the network learns to accumulate negative evidence and to use the pulses as excitatory triggers for producing the output spikes at the required times. Our results highlight the potential of spiking autoencoders as building blocks for more complex biologically-inspired architectures. We also provide open-source code for the model.

## 1. Introduction

Spiking neural networks (SNNs), hailed as the “third generation of neural networks” (Maass, 1997), are models of neuronal computation closely inspired by the biology of the brain. Conventional artificial neural networks (ANNs) are currently highly successful on a wide range of problems, routinely exceeding human performance (LeCun et al., 2015), but their representational capabilities lack one fundamental aspect characteristic of all biological organisms: the temporal dimension. While some ANN architectures, such as LSTMs (Hochreiter and Schmidhuber, 1997) and Transformers (Vaswani et al., 2017), can operate on sequential information with great results, encoding temporal information is not natural to ANNs. By contrast, in SNNs, neurons communicate through *spikes* fired at specific times, which adds an intrinsic temporal aspect to their information processing capabilities.

SNNs are of particular interest for the fields of neuromorphic hardware and computational neuroscience (Zenke et al., 2021). From the perspective of neuromorphic computing, SNNs provide models for event-based computing deployable in hardware, potentially with large energy savings compared to ANNs (Blouw and Eliasmith, 2020). As tools for neuroscience, they can bring insight into the computational capabilities of biological networks (Abbott et al., 2016).

One way of encoding data into spiking neural networks employs temporal coding, which posits that information is encoded in the *relative timing* of neuronal spikes. Further to its biological inspiration (which is detailed in section 4), temporal coding is of interest because it allows learning using exact gradients with respect to spike times, hence allowing the application of efficient standard machine learning techniques. This addresses one of the main obstacles that prevent a larger adoption of SNNs compared to ANNs, which is the difficulty of training the former. Spiking networks that employ temporal coding are sometimes called temporal neural networks (TNNs) (Smith, 2021). Notable existing work in this field includes the SpikeProp model (Bohte et al., 2000) and its extensions (Schrauwen and Van Campenhout, 2004; Booij and tat Nguyen, 2005; McKennoch et al., 2006; Ahmed et al., 2013; Wang et al., 2017; Mostafa, 2018; Hong et al., 2020). In previous work, we have shown that SNNs with temporal coding provide a class of universal approximators for well-behaved functions, and can be trained with backpropagaton to perform classification tasks to accuracies similar to ANNs (Comşa et al., 2021).

Autoencoders are a type of representation learning that was first introduced in the context of restricted Boltzmann machines for dimensionality reduction (Hinton and Salakhutdinov, 2006). Their applications include noise removal from corrupted data (Vincent et al., 2008) and generative modelling (Kingma et al., 2019) (see also Bengio et al., 2013; Goodfellow et al., 2016). They are interesting as a building block for deep learning and, more ambitiously, for architectures inspired by the human brain (Krauss and Maier, 2020). Spiking autoencoders have only been sparsely explored, for example by Roy et al. (2019). Unsupervised feature extraction with spiking neural networks using spike-time-dependent plasticity (STDP) has been shown to be feasible (Masquelier and Thorpe, 2007) and can be stacked with promising results (Kheradpisheh et al., 2018). It has been proposed that mirrored STDP implements autoencoder learning in SNNs (Burbank, 2015). However, it has also been argued that the performance of STDP learning can considerably lag behind that of conventional ANN autoencoders (Falez et al., 2019).

Here show that SNNs with temporal coding can learn to behave as autoencoders using standard backpropagation techniques. We characterise one-layer spiking autoencoders that learn to reconstruct images from the MNIST dataset of handwritten digits (LeCun et al., 1998), which we encode in an analog manner in the spike times, at multiple noise levels. We also verify that similar results are obtained on the Fashion-MNIST (FMNIST) dataset of clothing items (Xiao et al., 2017) encoded in the same way. Autoencoders are trained to reconstruct images with respect to multiple target latencies. We compare the performance of the SNN autoencoders with that of conventional ANN autoencoders, showing that they achieve at least similar performance. Further, we explore the embedding properties and the spiking dynamics of the autoencoders at different latencies and noise levels. We demonstrate that inhibition has an essential role when the input needs to be memorised for a longer time before the expected output spike times, whereas the learnable pulses are used as excitatory triggers for the target latency. These results establish SNN autoencoders as potential building blocks for more complex biologically-inspired architectures for neuromorphic computing. We provide open-source code for the model. We provide open-source code for the model at https://github.com/google/ihmehimmeli/tree/autoencoder/.

## 2. Methods

### 2.1. Spiking Neuron Model

We use a neuronal model previously described by Comşa et al. (2021). Upon spiking, an input neuron, indexed by *i*, produces an increase over time *t* in the temporal membrane of a downstream (output) neuron described by an α function (Sterratt et al., 2018) of the form $w_{i}\left(t-t_{i}\right)e^{-\tau \left(t-t_{i}\right)}$, where:

- *t*_*i*_ is the non-negative, real-valued spike time;
- *w*_*i*_ is the real-valued synapse efficiency, or weight;
- τ is a real-valued decay rate constant, fixed across the network, that scales the function in intensity and time.

**Figure 1:**
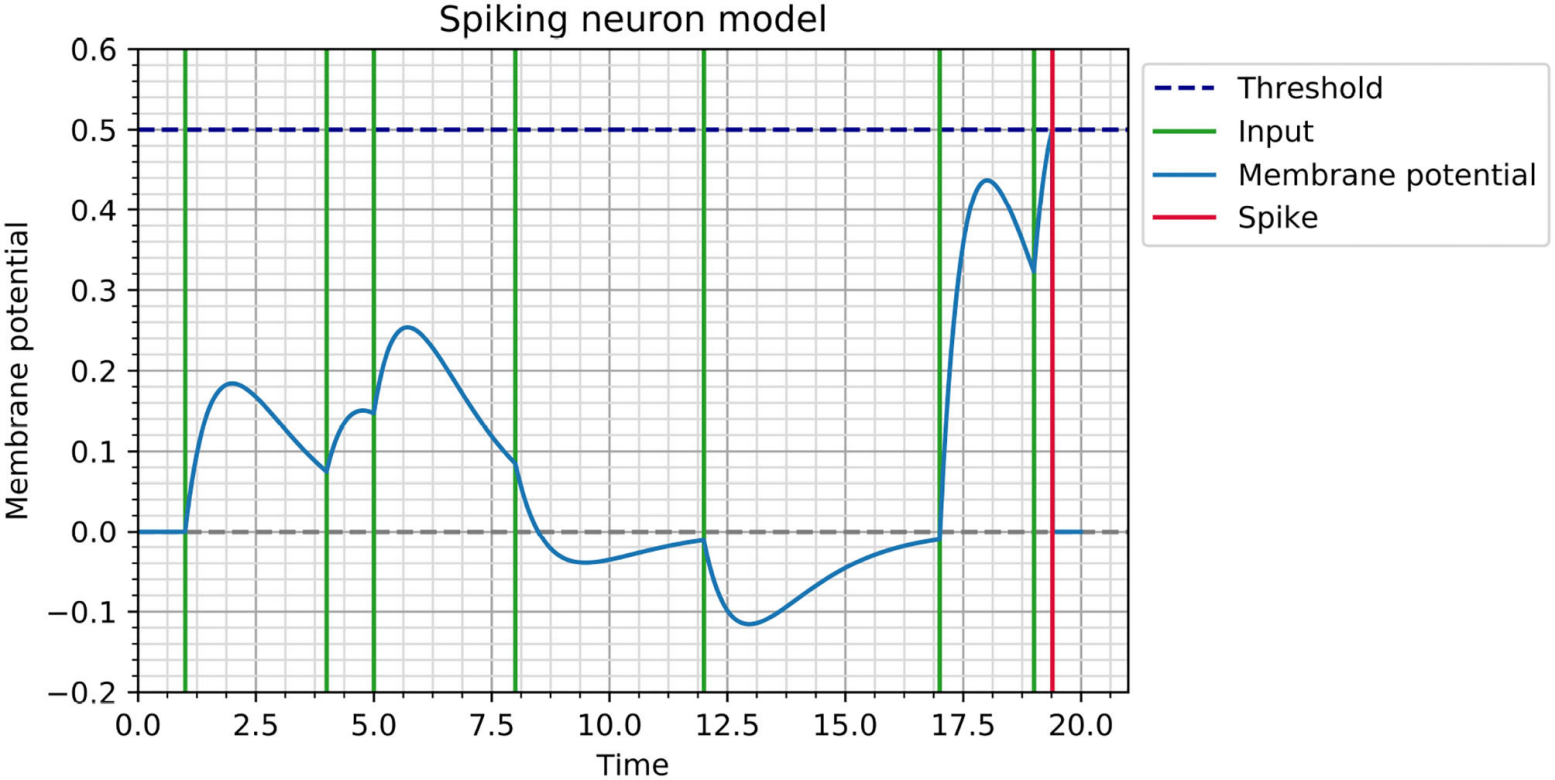
Illustration of membrane potential dynamics for a neuron with θ = 0.5 and τ = 1. The neuron receives input spikes at times *t*_*i*_ ∈ {1, 4, 5, 8, 12, 17, 19} with corresponding weights *w*_*i*_ ∈ {0.5, 0.3, 0.4, −0.2, −0.3, 1.2, 0.9}, which cause it to spike at *t*_*out*_ = 19.39.

This synaptic transfer function is inspired by recordings in biological neurons (Rall, 1967) and is illustrated in Figure 1.

Consider a neuron receiving a sequence of inputs *t*_*i*∈{1..*n*}_. Its membrane potential at any point *t* before spiking is given by $V\left(t\right)=\underset{i\in \left\{1..n\right\}}{\sum }w_{i}\left(t-t_{i}\right)e^{\tau \left(t_{i}-t\right)}$. As soon as its membrane potential reaches a threshold θ, which is fixed across the network, the neuron spikes and its membrane potential is reset. In the current feedforward autoencoder, any neuron spikes at most once, but this spiking neuron model and learning rule can also be used with multiple spikes per neuron and with recurrent architectures, by computing gradients corresponding to each event individually and combining them at each neuron. An implementation allowing for the construction of recurrent networks with multiple spikes can be found on the GitHub repository in the event_based branch.

On a regular computer architecture, simulating the spike times can be done in an event-based manner, without the need for discrete time steps. The spikes are processed one by one in chronological order and the membrane potential of any affected neuron is updated as required. One notable aspect of the simulation is finding the correct set of inputs that determine a neuron to spike; importantly, even if a set of input neurons determines the crossing of the threshold and hence predicts a future spike, this predicted spike may not occur, or may occur at a different time, if a new input spike comes between the last input spike and the predicted spike time.

Given a set of input spikes *t*_*i*∈{1..*n*}_ and their corresponding weights *w*_*i*∈{1..*n*}_, the output spike time is given by (refer to Comşa et al., 2021 for the full derivation):

(1) $$\begin{array}{r} t_{out}=\frac{B}{A}-\frac{1}{\tau }W_{k}\left(-\tau \frac{\theta }{A}e^{\tau \frac{B}{A}}\right) \end{array}$$

where $A=\underset{i}{\sum }w_{i}e^{\tau t_{i}}$, $B=\underset{i}{\sum }w_{i}e^{\tau t_{i}}t_{i}$ and *W*_*k*_ denotes branch *k* the Lambert *W* function (Lambert, 1758; Corless et al., 1996). This equation has two possible solutions corresponding to the ascending and the descending parts of the membrane potential function. We are interested in the earliest solution and therefore employ the main branch *k* = 0. The equation has exactly one solution if the maximum of the membrane potential is at θ, and no solution if θ is not reached and there is no spike.

### 2.2. Network Architecture

**Figure 2:**
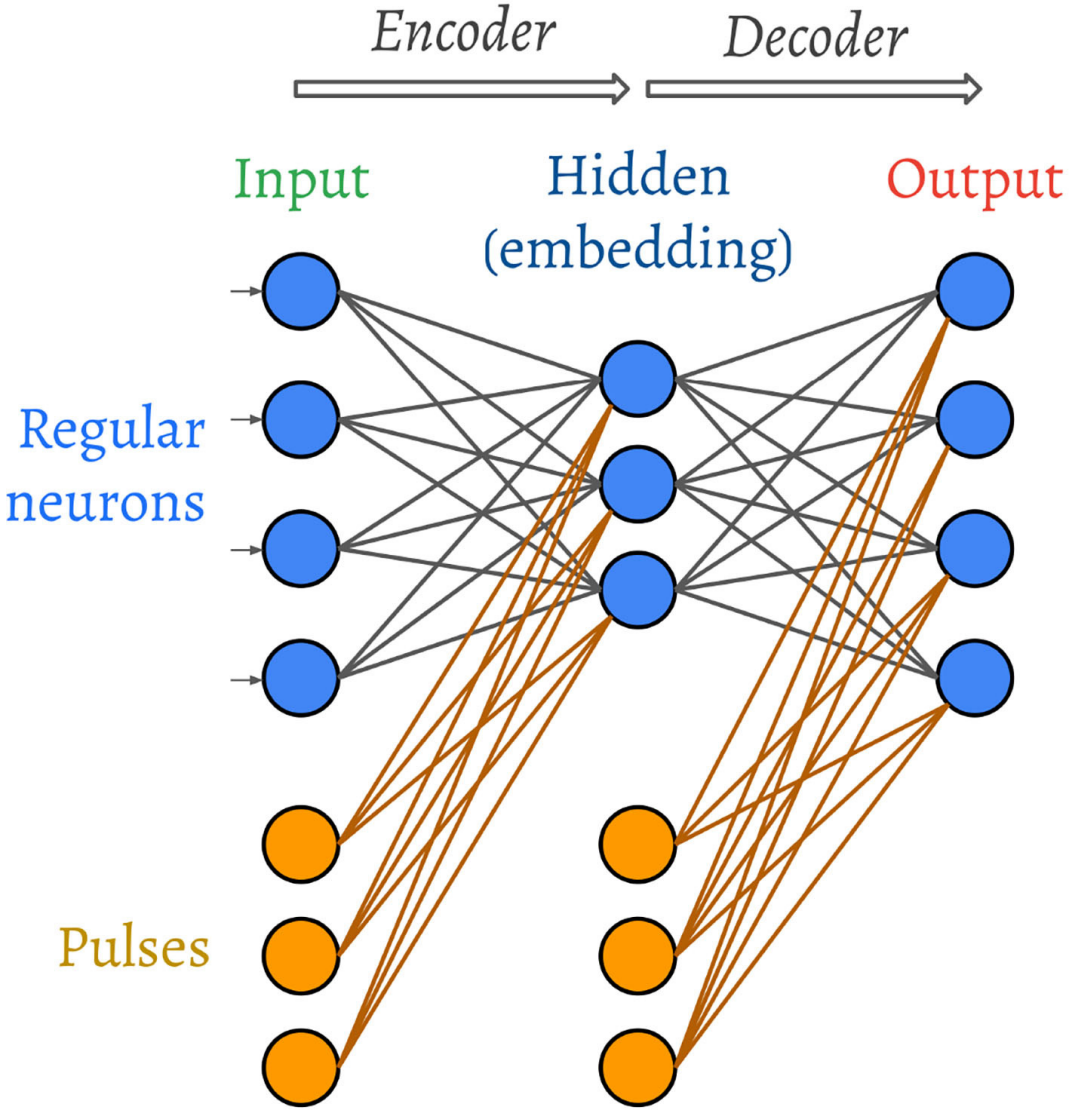
Architecture of the spiking autoencoder. The weights and the pulses are trainable.

The architecture of the spiking autoencoder is shown in Figure 2.

The autoencoder is composed of three layers: an input layer, a hidden layer that acts as an encoder and is composed of fewer neurons compared to the input layer, and an output layer that acts as a decoder and has the same size as the input layer. The purpose of the autoencoder is to learn to reproduce the input image in the output layer. In other words, the hidden layer must learn to convert the input image into a compressed representation, from which the output image can be reconstructed as closely as possible to the original.

For the MNIST problem, we use hidden layer sizes of *h* ∈ {8, 16, 32}. The size of an MNIST digit is 28 × 28 = 784, but the average number of non-empty pixels per image is around 150. Hence, the hidden layer sizes correspond to around {5, 10, 20%} of the original number of non-empty pixels per image in this dataset.

In addition to the regular neurons of the SNN, we also connect a variable number of “synchronisation pulses” to each neuron of each non-input layer. The role of the pulses, which can be thought of as non-input neurons, is to provide a temporal bias and encourage regular neurons to spike in order for gradients to keep flowing during the training process. Just like regular neurons, the pulses connect using learnable weights, but their spike times are also learnable under the same learning scheme as the rest of the network (as described in section 2.5). The set of pulses connected to each layer is initialised with pulses that spike at time evenly distributed in the same interval as the inputs. In section 3, we elaborate on the role that the pulses learn to play in the image decoding process.

### 2.3. Image Encoding

The temporal coding scheme posits that more salient information is encoded as earlier spike times. Given an image, we encode each of its individual pixels in the spike time of an individual neuron. The spike time is proportional to the brightness of the pixel. For example, *m* pixels with brightness levels in the interval [0, 1] can be encoded as the spike times of *m* neurons in the same interval, where a pixel with brightness of 0.1 corresponds to a spike time at *t*_*i*_ = 0.1.

In the original MNIST dataset (LeCun et al., 1998), all inputs are encoded as values in the interval [0, 1], but more salient information (the usually central pixels that encode the meaningful digit information in most images) is encoded as larger values. In this case, we invert the brightness of each image to obtain the spike times. White pixels (equal to 1 after brightness inversion) do not cause spikes, as it can be considered that they do not carry any information.

The idea of encoding more salient information as earlier spikes also appears in time-to-first-spike (TTFS) encoding schemes, which is often used in classification paradigms (Mostafa, 2018; Kheradpisheh and Masquelier, 2020; Sakemi et al., 2020; Zhang et al., 2020). However, since the objective of the autoencoder is image reconstruction, other encoding schemes could be used for the inputs, including an inverted scheme that we briefly explore in section 3.

### 2.4. Image Decoding

The aim of the decoder is to reproduce the input image from the compressed representation provided by the encoder. As the image is encoded in the spike times, we set a target latency *l*, which is fixed for the model, as a reference for the deconstruction. In other words, if an input pixel is encoded as a spike time at time *t*_*i*_, the target latency for the corresponding output pixel is *l* + *t*_*i*_. In this work, given the input interval [0, 1], we explore different models that aim to reconstruct the image with latencies *l* ∈ {1, 2, 4, 8, 16}. The target latency is directly encoded in the loss function (as described in section 2.5).

There exist alternative ways of choosing the temporal reference for decoding the output image. One possible alternative is to reconstruct with reference to the earliest spike in the output layer, which would give the SNN the freedom to self-regulate its spike times. Another possible way is to add an additional neuron to the output layer, which could explicitly act like a temporal reference. Here we opt for a fixed latency, which best allows us to study how the spike dynamics change as the model is required to wait for different times between producing the image reconstruction.

### 2.5. Learning Spike Times Using Backpropagation

The aim of training the spiking autoencoder is to obtain a faithful reconstruction of the input image at the output layer, with a given target latency *l*. We therefore minimise the following mean square error loss function:

(2) $$\begin{array}{r} L\left(t,\tilde{t}\right)=\overset{n}{\underset{i=1}{\sum }}{\left(t_{i}-\tilde{t_{i}}-l\right)}^{2} \end{array}$$

where *t* represents the original *n*-pixel input image (i.e., the *n* input spike times), and $\tilde{t}$ represents the *n*-pixel reconstruction (i.e., the *n* output spike times that represent the image produced by the SNN). If an output neuron does not spike, we use a very large value as a surrogate spike time, thus allowing the gradient to amplify the weights.

As in the case of conventional backpropagation training for neural networks, we use the chain rule to compute the update rules for each neuronal spike time and weight in the network by expanding the expression across *k* layers as follows:

(3) $$\begin{array}{r} \frac{\partial L}{\partial w_{j}}=\frac{\partial L}{\partial t_{j+k}}\frac{\partial t_{j+k}}{\partial t_{j+k-1}}...\frac{\partial t_{j}}{\partial w_{j}} \end{array}$$

By differentiating Equation (2), we can plug in the derivative of the loss function *L* with respect to the spike times in the output layer, which is simply $2\left(t-\tilde{t}-l\right)$. Next, to backpropagate the loss, we need to differentiate individual spike times *t*_*j*_ with respect to their direct input spike times *t*_*j*−1_ and their weights *w*_*j*_. Thankfully, the temporal coding scheme allows us to differentiate Equation (1) and obtain the exact derivatives of an output spike time *t*_*j*_ with respect to any input *t*_*j*−1_ and weight *w*_*j*_ (refer to Comşa et al., 2021 for the full derivation):

(4) $$\begin{array}{r} \frac{\partial t_{j}}{\partial t_{j-1}}=\frac{w_{j}e^{\tau t_{j-1}}\left(\tau \left(t_{j-1}-\frac{B}{A}\right)+W+1\right)}{A\left(1+W\right)} \end{array}$$

(5) $$\begin{array}{r} \frac{\partial t_{j}}{\partial w_{j}}=\frac{e^{\tau t_{j-1}}\left(t_{j-1}-\frac{B}{A}+\frac{W}{\tau }\right)}{A\left(1+W\right)} \end{array}$$

where *W* denotes the Lambert function $W_{0}\left(-\tau \frac{\theta }{A}e^{\tau \frac{B_{I}}{A_{I}}}\right)$.

We then plug these derivatives into Equation (3) to obtain the update quantities for each individual neuron and weight. Equation (4) can also be used for adjusting the spike times of the pulses. This is the same backpropagation procedure that is conventionally used in non-spiking ANNs.

If a neuron does not spike, then we add a small positive-valued penalty to each of the input weight derivatives, in order to encourage spiking. If an input neuron spikes after the output neuron, we do not compute derivatives corresponding to that input neuron or its weight.

As the derivative of each neuron can approach infinity when the membrane potential is close to the threshold θ, we clip the derivatives (Equations 4 and 5) during the training process using a fixed clipping value.

The training process consists of minimising the loss function, using an Adam optimiser, for 100 epochs. We use a modified form of Glorot initialisation (Glorot and Bengio, 2010) where the weights are drawn from a normal distribution with standard deviation $\sigma =\sqrt{2.0/\left({\text{fan}}_{in}+{\text{fan}}_{out}\right)}$ (as in the original scheme) and custom mean μ = multiplier × σ. We use different learning rates for the weights and for the pulses.

### 2.6. Noise Removal From Images

We train spiking autoencoders to reconstruct images under noisy conditions. We add normally distributed noise to each pixel in the following form, where η is the noise factor and *r* is a random variable drawn from a normal distribution with standard deviation 1:

(6) $$\begin{array}{r} t_{i}=\max\left(0,\min\left(t_{i}+\eta r,1\right)\right) \end{array}$$

The mean of the noise variable *r* is 1 in the case where the image brightness is inverted such that larger values in the input interval [0, 1] represent less salient information. If the image brightness is not inverted, the mean is set to 0.

We study spiking autoencoders trained on datasets with noise factors η ∈ {0, 0.2, 0.4, 0.6, 0.8}. The noisy images are used as training and test examples, while the training targets are the original (clean) images.

### 2.7. Hyperparameters

We have three variables controlling the setup for the spiking autoencoders: target latency *l* ∈ {1, 2, 4, 8, 16}, noise factor η ∈ {0, 0.2, 0.4, 0.6, 0.8}, and embedding size *h* ∈ {8, 16, 32}. For each combination of these parameters, we do a search to find the best set of hyperparameter controlling the model and the training options. The hyperaparameter search is conducted using the Google Vizier framework (Golovin et al., 2017) using evolutionary-neural hybrid agents (Maziarz et al., 2018), with minimum of 1,000 and up to 7,000 trials per condition. The search is conducted on the MNIST datasets only and the best configurations are then used on FMNIST as well.

For each model analysed below, we report results obtained with the best hyperparameter combination for its setup. However, we can find sets of parameters with good performance on multiple setups at each target latency. These parameters are shown in Table 1. The hyperparameters used for each model described in this paper can be found in the GitHub repository.

**Table 1:** Hyperparameters that achieve an error within 0.01 of the best error on all the training configurations with the given latency.

**Hyperparameter**	**Search range**	**Value (*l* = 1)**	**Value (*l* = 16)**
decay_constant (τ)	[0.1, 2]	0.3138976904122206	0.28781361955998486
fire_threshold (θ)	[0.1, 1.5]	0.8011900124783229	0.9063259346518524
n_pulses	[0, 10]	10	8
nonpulse_init_multiplier	[−10, 10]	−9.533865719823941	−6.971635832107275
pulse_init_multiplier	[−10, 10]	−8.08055538136939	9.978394158917038
batch_size	[1, 1000]^*^	3	27
clip_derivative	[1, 1000]	247.36488789120077	373.3754658744521
penalty_no_spike	[0, 100]	33.83286251355259	39.560790380375444
learning_rate	[10^−5^, 1.0]^*^	0.0016762843980764315	0.00038521130189147893
learning_rate_pulses	[10^−5^, 1.0]^*^	0.0014413603337483233	0.13300674961971326

*The best set of hyperparameters for each individual {latency, noise, embedding size} configuration can be found on the GitHub repository*.

* *Logarithmic search space*.

### 2.8. ANN Baseline

We compare spiking autoencoders with conventional ANN (non-spiking) autoencoders of similar architecture. Specifically, a single hidden layer of hidden size 8, 16, or 32 is used, acting as the encoder. We use ReLU activation function in the encoder and a sigmoid activation function in the decoder (but see the Results for a brief exploration of other activation functions).

The ANNs are implemented in TensorFlow (Abadi et al., 2015). Note that the ANN autoencoders do not have pulses, but they have bias terms, which leads to a slightly smaller parameter count in the ANN autoencoders [the number of parameters of a SNN with *p* pulses and an architecture of the form *i* − *h* − *o* is equal to (*i*+*p*)*h+(*h*+*p*)*o+2*p; comparatively, the number parameters in an ANN with biases is i*h+*h*+h*o+*o*]. We use the same optimiser and number of epochs for both the ANN and the SNN autoencoders.

The input MNIST images are inverted in order to be fed into the spiking autoencoders, given that more salient information causes earlier spikes, so that the more central pixels should cause spikes closer to *t* = 0 compared to background pixels. In contrast, the natural representation of a MNIST in a conventional ANN is the original (not inverted) version. We start by comparing these two representations, but later also explore inverting this representation in both the spiking and the conventional networks.

## 3. Results

### 3.1. Reconstruction Loss

**Figure 3:**
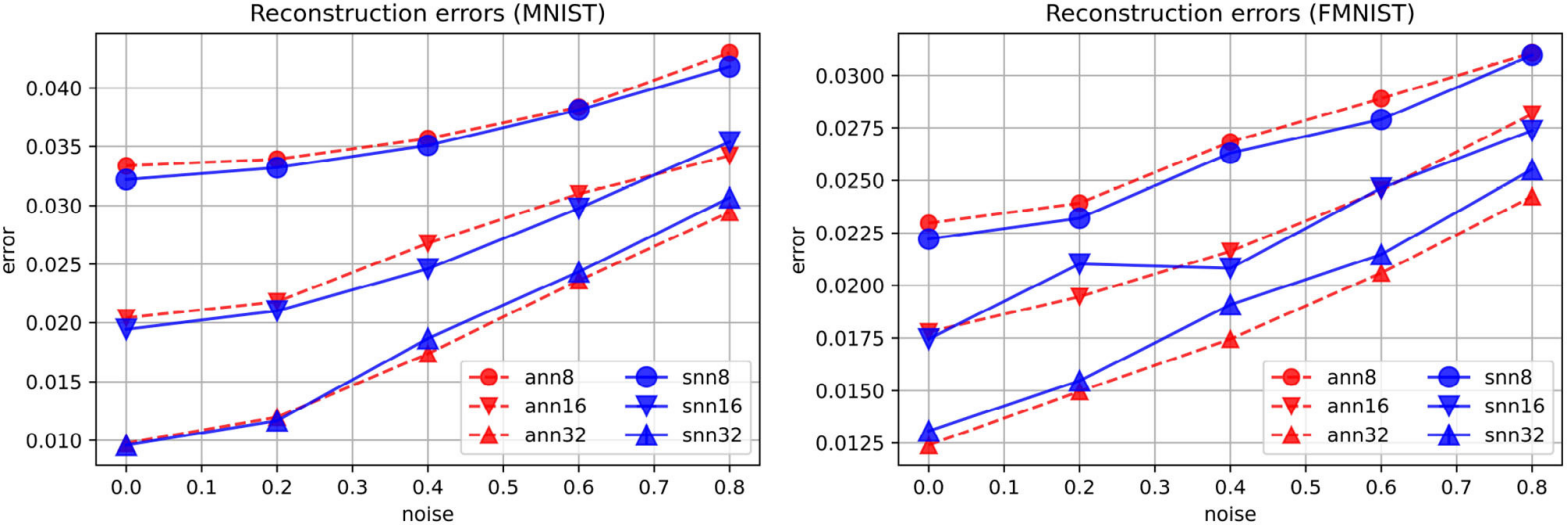
Reconstruction errors for spiking (“snn”) and non-spiking (“ann”) autoencoders at different levels of noise, for embedding sizes 8, 16, and 32, on the MNIST and FMNIST datasets.

The best reconstruction loss values obtained in the spiking autoencoders and the ANN autoencoders are similar (Figure 3). In the spiking autoencoder, we find that the noise level and the embedding size have a large effect for both the spiking and the ANN autoencoders.

**Figure 4:**
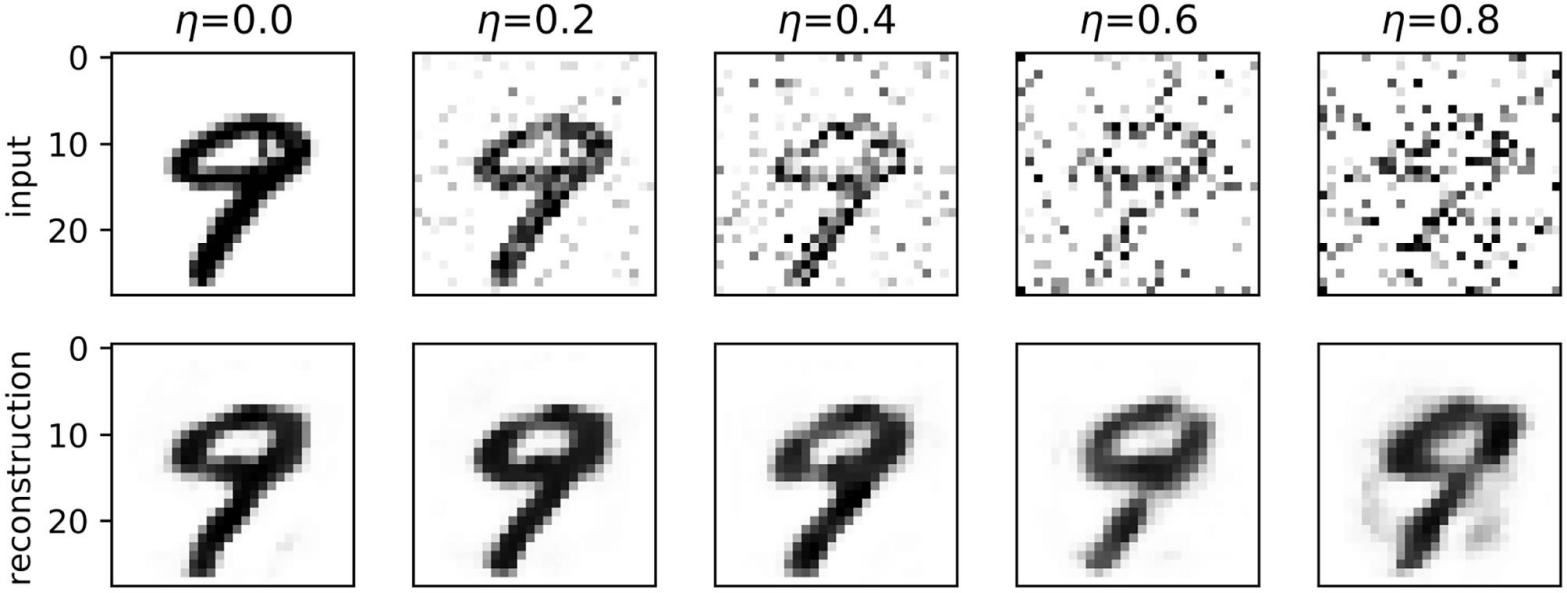
A digit from the MNIST test set reconstructed by a spiking autoencoder with embedding size 32 and target latency *l* = 1, at different levels of noise.

Figure 4 shows examples of reconstructions produced by spiking autoencoders trained at different noise levels and embedding sizes, demonstrating the loss of quality with more noise and smaller embeddings. Nevertheless, the spiking autoencoder is able to reconstruct original images from highly noisy images relatively well. Similar results are obtained for ANN autoencoders.

### 3.2. Embedded Features

**Figure 5:**
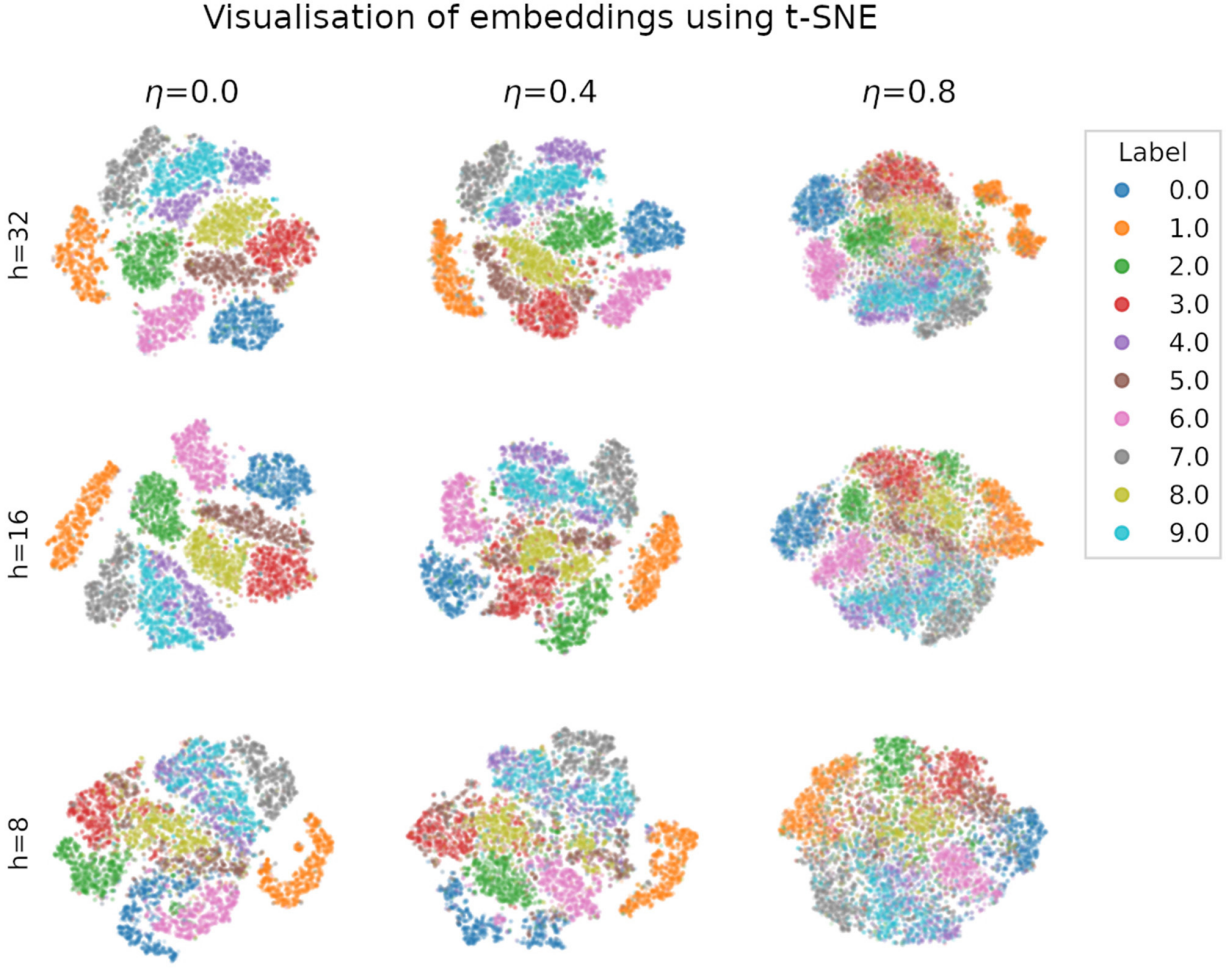
Visualisation of MNIST embeddings produced by a spiking autoencoders with target latency *l* = 1 at different levels of noise η and embedding sizes *h*, using the t-distributed stochastic neighbour embedding (t-SNE) technique, with perplexity set to 20. The results are qualitatively similar for different perplexity values. Axis units (not shown) are arbitrary and identical for each plot.

We visualise in Figure 5 the quality of the embeddings produced by spiking autoencoders. We use t-distributed stochastic neighbour embedding (t-SNE) (van der Maaten and Hinton, 2008), a method that assigns 2-dimensional coordinates to high-dimensional points based on their similarity. The t-SNE algorithm was initialised using principal component analysis and run with perplexity value of 20. We verified that the results were similar for multiple perplexity values. The relative distance between digit clusters decreases with higher noise level and smaller embeddings size. Similar results are obtained for ANN autoencoders.

**Figure 6:**
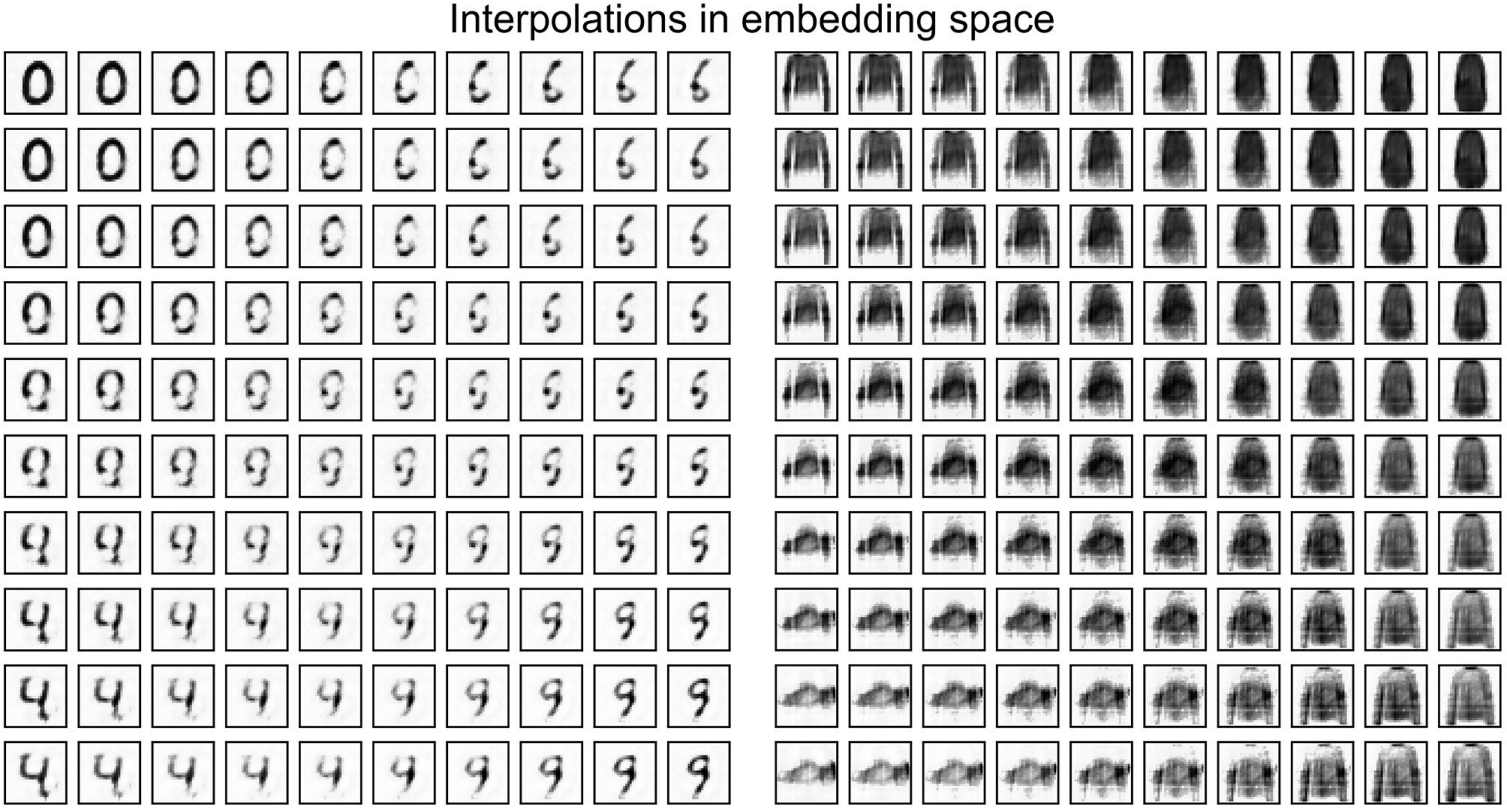
Interpolating between four items from the MNIST and FMNIST test sets in embedding space. The embeddings are generated by a spiking autoencoder with hidden layer size 32, target latency *l* = 1, noise level η = 0. They are then interpolated and, finally, run through the decoder layer to obtain the representation in original space.

A practical use of embeddings comes from collapsing a high-dimensional input space, from which the training distribution is sparsely drawn, into a smaller space where basic operations like addition are meaningful. Figure 6 shows the interpolation between four digits in original space and in embedding space, demonstrating meaningful digit-like intermediary steps only when the interpolation is done in embedding space. Similar results are obtained using ANN autoencoders.

**Figure 7:**
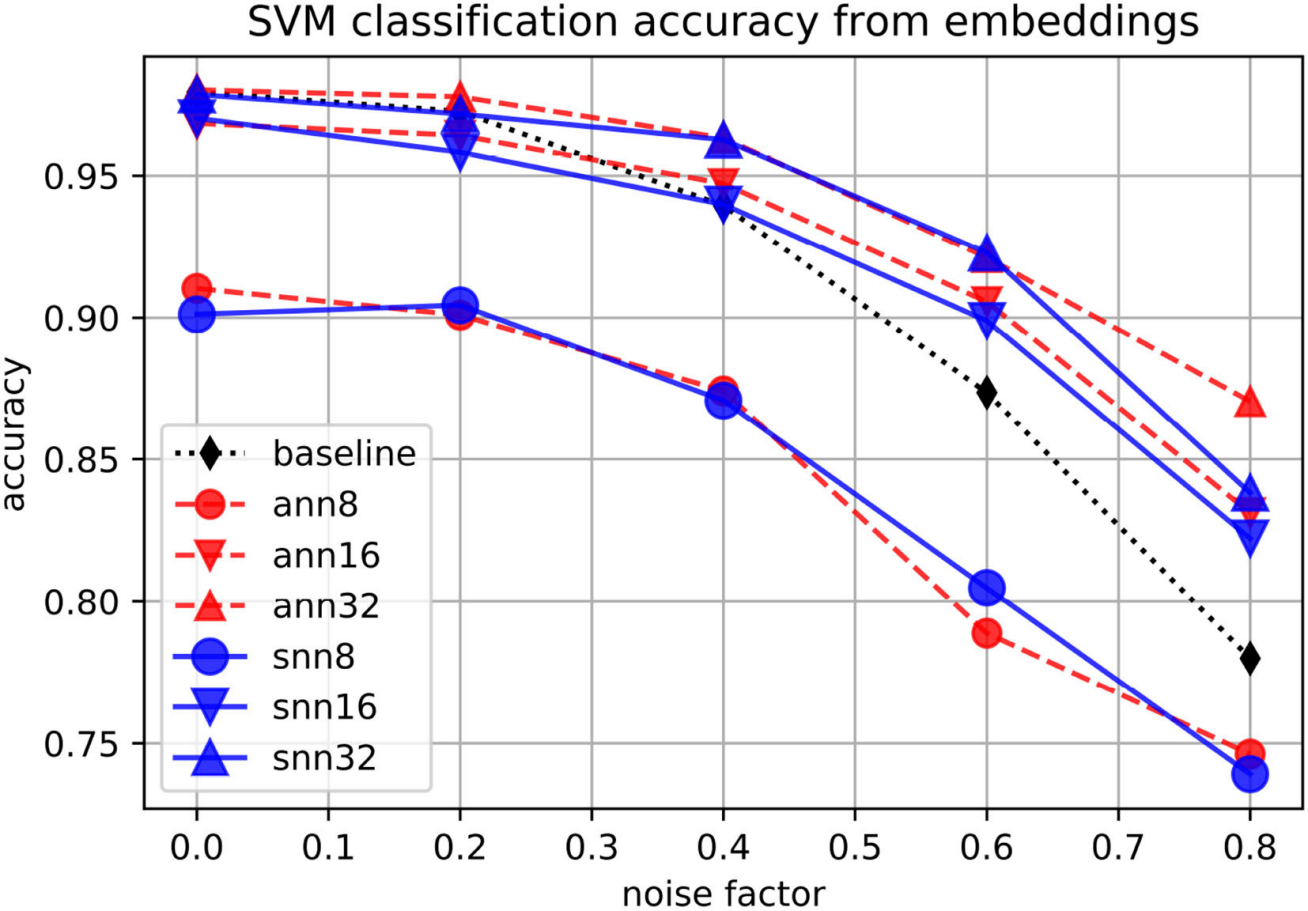
Accuracy of an SVM classifying embeddings produced by spiking (“snn”) and non-spiking (“ann”) autoencoders at different levels of noise, for embedding sizes 8, 16, and 32, on the MNIST dataset. The baseline is the classification accuracy on the original set.

Finally, we use support-vector machines (SVMs) with Gaussian kernel to classify digits using either the original space or the embeddings as input features, at different levels of data noise. As shown in Figure 7, in the noisier conditions, the classification accuracy is higher when embeddings of size at least 16 are used as classification input features. The performance for ANNs and SNNs is similar, with neither of them consistently outperforming the other.

### 3.3. Spike Dynamics and Weight Distributions

Having established that spiking autoencoders with temporal coding perform on par with their ANN counterparts qualitatively and quantitatively, we proceed to a more in-depth analysis of the trained SNN models. These analyses are performed on the MNIST dataset.

**Figure 8:**
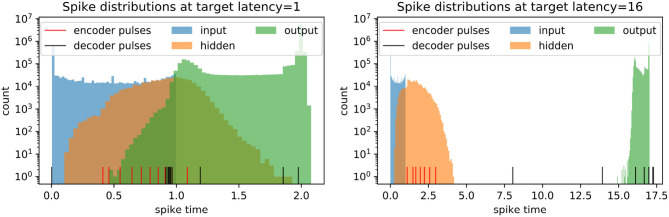
Spike distributions on the full test set in trained spiking autoencoders with embedding size 32, noise level η = 0, target latencies *l* = 1 and *l* = 16. The pulses are shown individually.

We investigate the models with different spike latencies trained to reconstruct original images (no noise). Intriguingly, we find that the distribution of the embedding (hidden layer) spikes does not shift away from the input distribution and toward the output distribution with higher target latency, but rather remains relatively early, as shown in Figure 8. In contrast, the pulses shift toward later times with higher target latency. This suggests that the pulses play an excitatory role at higher latency, acting like triggers for eliciting spikes with the required delay.

**Figure 9:**
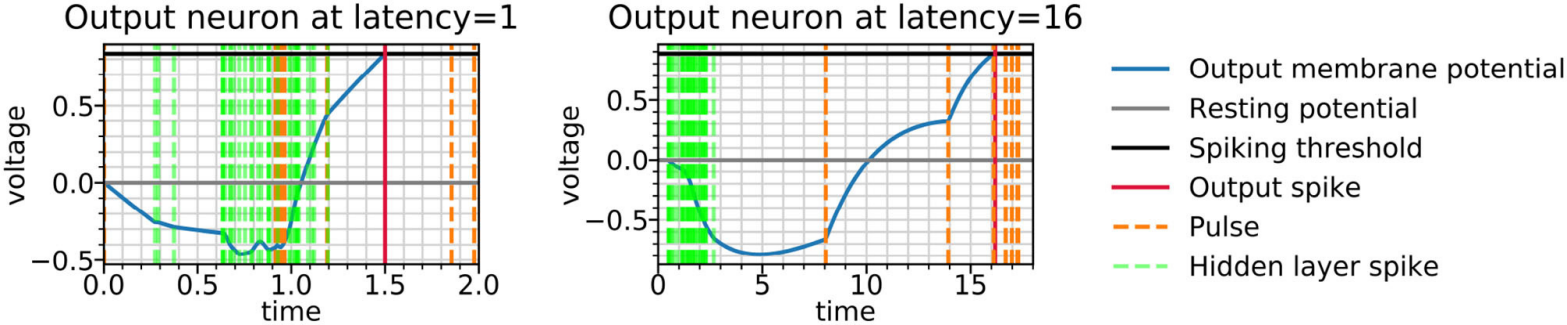
Output potentials during the reconstruction of a test example by spiking autoencoders with embedding size 32, noise level η = 0, target latencies *l* = 1 and *l* = 16. The output neuron is chosen such that the target spike time is smaller than *l* + 0.1 (in other words, it is located in the centre of the image and encodes salient digit information). The figure underlines the initial negative response of the membrane voltage, followed by a positive response caused by pulses.

**Figure 10:**
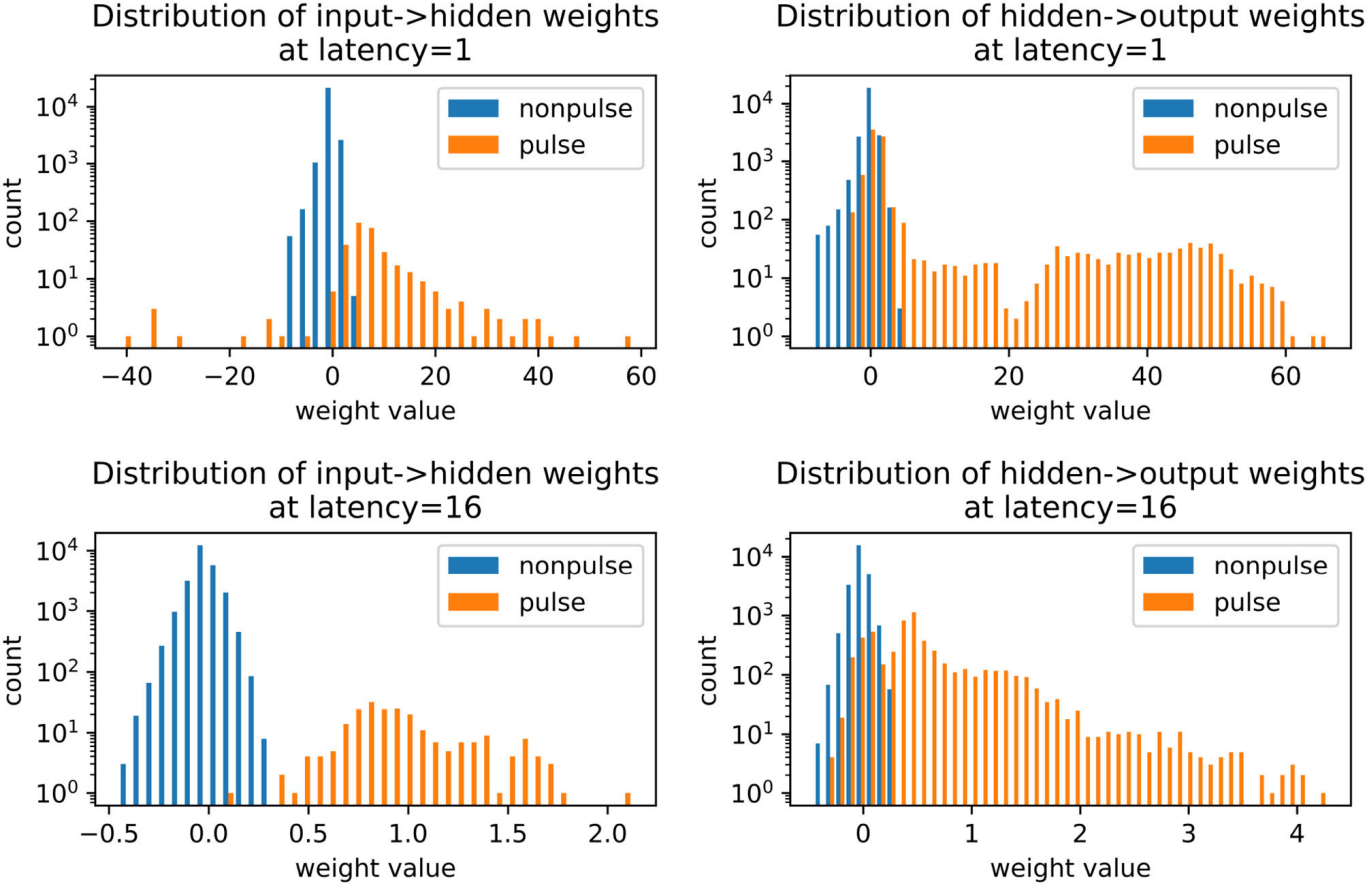
Weight distributions in spiking autoencoders, for regular neurons and pulses. All models have embedding size *h* = 32 and noise level η = 0.

The role of inhibition at the higher latency can be more directly observed in Figure 9, where the membrane potential dynamics in the output neurons indicate that the neurons are inhibited by the incoming spikes from the hidden layer. The output spike is triggered by pulses, which occur closer to the target spike time and have a strong excitatory effect. Examining the weight distributions for the regular neurons and the pulses in the encoder and the decoder in Figure 10, it is clear that regular neurons have on average inhibitory weights, whereas pulses are strongly excitatory. As this happens in both layers of the network, this can be interpreted as accumulating negative evidence for inhibition.

### 3.4. ANN Performance on Inverted-Value Images

As mentioned in section 2, the default input to the temporally-coded SNN is the inverted version of the images (*p*: = 1 − *p*), such that the more informative pixels have values closer to 0, whereas the less informative pixels have values closer to 1. The ANNs presented so far were trained, as conventional, on the non-inverted images. We briefly explored how they would perform on the inverted version of the images.

**Figure 11:**
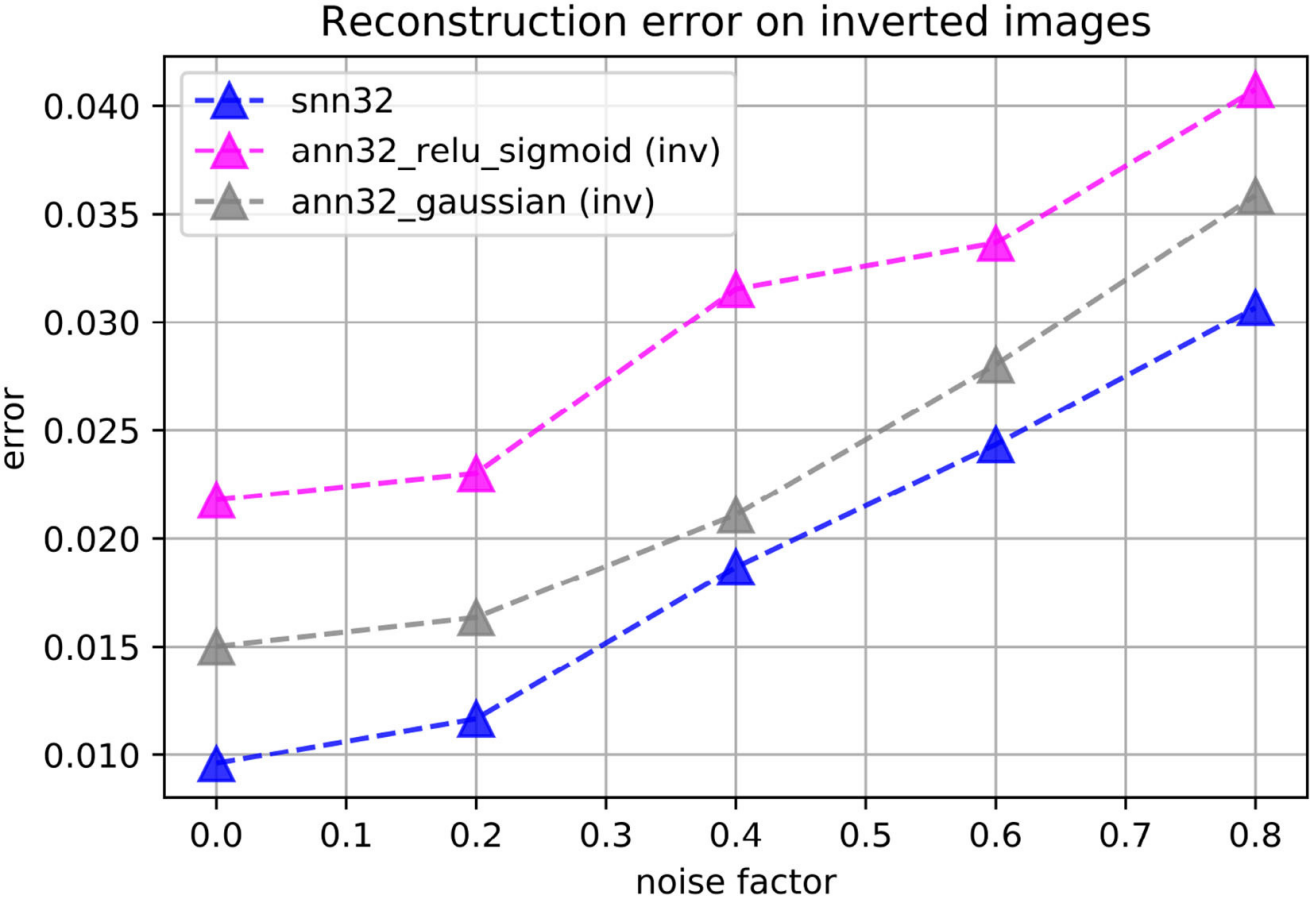
Reconstruction loss on the inverted-brightness MNIST dataset for spiking (“snn”) and non-spiking (“ann”) autoencoders. The embedding size is always *h* = 32. The spiking autoencoder has a target latency of *l* = 1. The non-spiking networks have either ReLU activation functions in the encoder and sigmoid activation functions in the decoder, or zero-centred Gaussian-like activation functions everywhere.

We found that ANN autoencoders do not perform as well at reconstructions on the inverted version of the images. We explored multiple choices of activation function combinations, including ReLU, sigmoid, ELU, tanh, and Gaussian-shaped functions in either the encoder of the decoder. The best performing ANN autoencoders had Gaussian-Gaussian and ReLU-sigmoid activation functions in the encoder and decoder layers, respectively. As shown in Figure 11, the spiking autoencoders outperform all the tested ANNs on the inverted images. Additional processing of the input dataset may help ANNs achieve better performance; as our aim is to study spiking autoencoders without additional data processing, we do not investigate further.

## 4. Discussion

We have shown that spiking autoencoders that represent information in the timing of neuronal spikes can learn to reconstruct images through backpropagation learning. They perform on par with conventional artificial neural networks, and exceed their performance when the inputs are encoded such that the smaller values correspond to more salient information. We have illustrated the capabilities of spiking autoencoders through multiple examples, and we have underlined the important role of inhibition especially when the SNN is required to keep the information in memory (i.e., in the membrane potential of the spiking neurons) for a longer time.

### 4.1. Biological Considerations

We discuss here the choice of coding scheme in a spiking network in relation to biology of the brain, as well as some considerations on backpropagation.

There are multiple ways of encoding information in the form of spikes. Very often, information is encoded in the neuronal spike rates. In such coding schemes, a more salient stimulus is encoded as a higher spike rate of a particular neuron. ANNs can, in fact, be thought of as operating with neuronal spike rates averaged in time or in space. While rate coding has practical value for comparisons with currently spatially-constrained methods of neural recording (Yamins and DiCarlo, 2016), it is arguably not optimal (Rullen and Thorpe, 2001). Overall, it is not clear that spike rates may best reflect the true computational substrate of the brain (Brette, 2015).

On the other hand, the idea of temporal coding is supported by multiple pieces of biological evidence, in particular at sensory-level such as in the retina (Gollisch and Meister, 2008) or in the tactile system (Jones et al., 2004; Johansson and Flanagan, 2009), but also in the higher in the neural processing hierarchies (Reinagel and Reid, 2000). The short time needed for visual stimuli to produce discriminating signals in the temporal lobe implies that single spikes must play an important rule in the propagation of information across the brain (Thorpe and Imbert, 1989). Our work hence encourages the use of temporal coding as a plausible information coding scheme for sensory processing in biologically-inspired neural models.

In this work, we used backpropagation to teach SNNs to reconstruct and remove noise from images of handwritten digits. The idea of backpropagation learning occurring in the biological brain is often questioned. However, it has been shown that random connections (Lillicrap et al., 2016) or other simple learning rules (Bengio et al., 2015) may approximate backpropagation-like learning. Here we do not claim that the learning method is necessarily biologically-plausible, but rather effective as a learning algorithm for training networks with other biologically-inspired characteristics.

### 4.2. The Role of Inhibition

A finding of particular interest that emerges from this work is the interplay between inhibitory and excitatory connections in producing spikes with the required timing. We allowed each connection to learn its own weight, without fixing its polarity from the beginning, but we allowed the hyperparameter search to influence the initial distribution of weights in the pulses and in the regular neurons. The networks learned to use inhibition as a main mechanism in the regular neurons, whereas the pulses were used as excitatory triggers to elicit output spikes at the target latencies. We remark that inhibition was used in both the hidden (encoder) layer and in the output (decoder) layer, which suggests a double accumulation of inhibition; in other words, the encoder accumulated information about which inhibitory elements should be inhibited in the decoder. This feature was not hard-coded in the models. As a consequence, the output timing of the network can be adjusted by simply changing the timing of the pulses connected to the output layer.

Despite our model being an oversimplification over the many variables observed in real neurons, it is still relevant to note that the balance of inhibition and excitation is essential in producing the spike patterns routinely observed in biological networks. For example, the theta rhythm in the hippocampus, which modulates memory, is thought to be caused by an intricate play between excitatory and inhibitory sources (Buzsáki, 2002). Simulations show that Poisson distributions of spikes, which often appear to be random in nature, may actually reflect the interactions between excitatory and inhibitory inputs with slightly different phase characteristics (Denève and Machens, 2016). Our model offers a simple demonstration of how negative evidence accumulation of inhibition, coupled with excitatory self-regulation, may be used to solve an image reconstruction task with externally-imposed timing.

### 4.3. Limitations

A significant challenge in scaling the current model and learning scheme is the computational demand during both the feedforward pass and the error backpropagation. Our model uses a synaptic function that has the advantage of being biologically faithful at the expense of requiring the computation of multiple exponentials. Moreover, a general drawback of spiking neural networks is that the event-based nature of the computation does not allow for full parallelisation in non-specialised hardware. In practice, our models can take a couple of minutes per epoch to train on regular hardware. Nevertheless, we have verified that deeper spiking autoencoders with up to five total layers can successfully learn the same datasets, although we chose to present here only single-layer experiments, which already achieved acceptable reconstructions. Convolutional variations are also possible, but they pose the same computational challenges on regular hardware.

### 4.4. Conclusions and Outlook

Our work accrues evidence for the potential of spiking neural networks with biologically-inspired characteristics as building blocks for neuromorphic machine learning. The novelty of our work consists in showing for the first time that spiking autoencoders with temporal coding can achieve results on par with conventional autoencoders, as well as providing insights into their dynamics, including the important role of inhibition in memorising information over longer periods of time. The inhibition across the network is complemented by the excitatory role that pulses learn to play in order to trigger the network output at the required time. Further to single-layer architectures, autoencoders can be stacked (Vincent et al., 2010) and even used for architectures inspired by the human brain (Krauss and Maier, 2020). Our model can thus be used as a building block for spiking neural networks with more complex architectures and energy-efficient neuromorphic computing.

## Data Availability Statement

The original contributions presented in the study are included in the article/supplementary material, further inquiries can be directed to the corresponding author/s.

## Author Contributions

IMC designed and performed the experiments, wrote the code, and wrote the first draft of the manuscript. LV contributed to data analysis, performance optimisation and code reviews, and led the open-sourcing. TF contributed to the conception of the study, literature survey, and code reviews. JA contributed to the conception, design, and resource availability for the study. All authors contributed to manuscript revision, read, and approved the submitted version.

## Conflict of Interest

All authors were employed by Google Research, Switzerland. Parts of the ideas presented here are covered by pending PCT Patent Application No. PCT/US2019/055848 (Temporal Coding in Leaky Spiking Neural Networks), filed by Google in 2019.

## Publisher's Note

All claims expressed in this article are solely those of the authors and do not necessarily represent those of their affiliated organizations, or those of the publisher, the editors and the reviewers. Any product that may be evaluated in this article, or claim that may be made by its manufacturer, is not guaranteed or endorsed by the publisher.
